# Energy metabolism and maternal-fetal tolerance working in decidualization

**DOI:** 10.3389/fimmu.2023.1203719

**Published:** 2023-06-19

**Authors:** Xinhang Meng, Chunqin Chen, Jinfeng Qian, Liyuan Cui, Songcun Wang

**Affiliations:** Laboratory for Reproductive Immunology, Hospital of Obstetrics and Gynecology, Fudan University Shanghai Medical College, Shanghai, China

**Keywords:** decidualization, energy metabolism, maternal-fetal crosstalk, decidual stromal cells, decidual immune cells, trophoblast cells

## Abstract

One pivotal aspect of early pregnancy is decidualization. The decidualization process includes two components: the differentiation of endometrial stromal cells to decidual stromal cells (DSCs), as well as the recruitment and education of decidual immune cells (DICs). At the maternal-fetal interface, stromal cells undergo morphological and phenotypic changes and interact with trophoblasts and DICs to provide an appropriate decidual bed and tolerogenic immune environment to maintain the survival of the semi-allogeneic fetus without causing immunological rejection. Despite classic endocrine mechanism by 17 β-estradiol and progesterone, metabolic regulations do take part in this process according to recent studies. And based on our previous research in maternal-fetal crosstalk, in this review, we elaborate mechanisms of decidualization, with a special focus on DSC profiles from aspects of metabolism and maternal-fetal tolerance to provide some new insights into endometrial decidualization in early pregnancy.

## 1 Introduction

Decidualization is histologically characterized by the appearance of larger and rounder cells surrounding the spiral arteries (SAs) and eventually spreading most of the endometrium. The transition from the proliferative stage to the secretory stage of the endometrium leads to the differentiation of endometrial stromal cells (EnSCs) into a special cell type called decidual stromal cells (DSCs) when EnSCs widely proliferate, getting larger and rounder with more changes in nuclear morphology and phenotype (1–3). DSCs secrete more prolactin (PRL), insulin-like growth factor binding protein 1 (IGFBP1), neuropeptide and extracellular matrix than EnSCs, among which PRL and IGFBP1 are extensively regarded as phenotypic markers of decidualization (4). DSCs are a distinct cell type resulting from terminal differentiation and genetic reprogramming of EnSCs, including downregulation of pro-inflammatory genes, as well as upregulation of genes that facilitating cellular proliferation, maternal-fetal tolerance and tissue invasion (5). These transformations further establish a proper environment for the subsequent placental formation and fetal development. Decidualization results from a complex and well-orchestrated differentiation program that involves all cellular elements of the mucosa: stromal, glandular and immune cells (6). Broadly defined, decidualization also includes accumulation of local immune cells, secretory changes of the uterine glands, SA remodeling, and extracellular matrix reconstruction (7).

In human, decidualization occurs in the luteal phase during each menstrual cycle (“pre-decidualization”), induced by the ovarian steroid hormones after ovulation regardless of fertilization or pregnancy, and continues after implantation (“decidualization”) (8). However, decidualization might vary in different physiological contexts. In the menstrual cycle, decidualization is characterized by direct communication between lymphocytes and stromal fibroblasts and a moderate rise in cytotoxic potential of lymphocytes. While during pregnancy, decidualization is tightly correlated with dynamics of window of implantation (WOI). It initiates before the opening of the WOI in a fraction of stromal fibroblasts and spreads wider at the receptive state (9). 17 β-estradiol plus progesterone (P4) or cyclic adenosine monophosphate (cAMP) plus medroxyprogesterone acetate (MPA) are always used to stimulate decidualization *in vitro (* 10, 11). Despite the cardinal endocrine mechanism by 17 β-estradiol and P4, as well as some other hormones like prostaglandins (PGs), luteinizing hormone (LH), follicle stimulating hormone (FSH), human chorionic gonadotropin (HCG) and gonadotropin-releasing hormone (12–15), metabolic and epigenetic regulation are also proved to be involved in this process recently.

In mice, the receptive state of uterus maintains for a short time, from gestation day (Gd) 4 to the afternoon of Gd 5 (16). Consistently, embryos enter the uterine cavity at Gd 3.5 and attach to the endometrium at Gd 4 (17). Subsequently, decidualization is triggered by the attachment and relies on the embryo implantation at Gd 4.5 to support embryo development (16–18). Artificial stimulation like oil infusion and uterine scratch can also induce decidualization when applied locally to pseudo-pregnant mice (14, 15). At Gd 5.5, decidualization initiates from stromal cells surrounding SAs, and forms a zone called the primary decidual zone (PDZ). Stromal cells next to PDZ continue to proliferate and differentiate into DSCs of the secondary decidual zone at Gd 7.5. They spread throughout the whole endometrium accompanied by trophoblast invasion (2, 12, 18).

Appropriate endometrial decidualization is essential for a successful pregnancy. The decidua offers a nutritive and immune-privileged microenvironment critical to embryo implantation, placentation, trophoblast invasion, immunomodulation and maintenance of pregnancy (19, 20). Shu-Wing Ng et al. proposed that the foundation of a healthy pregnancy is laid at the time of endometrial decidualization, prior to the establishment of pregnancy, just like that fertile and nontoxic soil is an essential premise for the growth of seeds (5). Impaired decidualization may cause numerous pregnancy disorders, such as infertility, recurrent spontaneous abortion (RSA), *in vitro* fertilization (IVF) failures, intrauterine growth restriction, preeclampsia, premature labor and so on (21–23).

Both in humans and mice, decidualization is continuous and consistent with trophoblast invasion. The underlying mechanisms are still not clear to date. Although most studies related to decidualization focus on the differentiation of DSCs, which are the major cellular component of human decidua, the recruitment and education of decidual immune cells (DICs), and the crosstalk among DSCs, DICs and trophoblasts are also vital during decidualization. Recently, dysregulation of metabolism in various physiological and pathophysiological courses, such as inflammation, angiogenesis, and cancer, are of great concern, and also have been proved to be related to decidualization (24). In this review, we will summarize the crosstalk of functional cells at the maternal-fetal interface during decidualization with a special focus on the metabolic and immune regulation on the initiation and maintenance of decidualization.

## 2 Energy metabolism reprogramming in DSC differentiation

Metabolism is regarded as a biological process of matter and energy exchange compose of a series of orderly chemical reaction to sustain biological activities, which enables organisms to grow, reproduce, maintain their structures and respond to the surrounding environment. Conceivably, decidualization is an energy-expensive process for its multistep processes accompanied by significant SA remodeling and extracellular matrix reconstruction (25). Energy metabolism reprogramming, including carbohydrate metabolism, lipid metabolism and amino acid metabolism, are proved to be involved in DSC differentiation.

### 2.1 Carbohydrate metabolism and DSC differentiation

Glucose utilization in the endometrial stroma is obviously increased upon exposure to P4. While the failure of decidualization and the subsequent embryo implantation may be the results of improper glucose uptake or carbohydrate metabolism in EnSCs (26, 27). The glucose metabolism initiates from an adequate intracellular glucose uptake, which is mediated by a family of glucose transporter (GLUT). The GLUT family has eight isoforms, GLUT1-GLUT8, characterized by the presence of 12 membrane-spanning helices and several conserved sequence motifs (28). In human endometrium, only GLUT1 and GLUT3 can be detected. Unlike the expression pattern of GLUT3, which is changeless in all studied cell segments throughout the decidua but especially higher in cluster of differentiation (CD)45 positive leukocytes, expression level of GLUT1 stays low in the proliferative stage but significantly increases in secretory phase and in 6-9 weeks of gestation (27). An *in vitro* experiment also showed that GLUT1 and glucose-6-phosphate dehydrogenase (G6PD) were highly upregulated in decidualizing human EnSCs (HEnSCs) than non-decidualizing ones, while there was no difference in GLUT3 expression between them (29). GLUT1 knockdown impairs glycolysis and HEnSC decidualization by reducing the mRNA levels of decidualization markers (IGFBP1 and PRL) and aerobic glycolysis-related genes (lactate dehydrogenase A (LDHA) and monocarboxylate transporter 4 (MCT4)), decreasing glucose uptake and lactate production, as well as activating apoptotic pathways (30). GLUT1 is also found as a functional protein cargo carried by endometrial stromal extracellular vesicles (EVs). Addition of EVs to HEnSCs showed an increased GLUT1 protein level without a concomitant increased mRNA level, reflecting the incorporation of EVs with GLUT1 cargo proteins directly into the HEnSC membrane. The internalization of EVs carrying GLUT1 promotes glucose uptake in recipient HEnSCs, resulting in boost in energy metabolism and acceleration in decidualization program (31). Overall, stromal GLUT1 may exert a critical role in decidualization and the preparation of blastocyst implantation (32–34).

Glucose serves as a metabolic signal for decidualization. Low-glucose environment suppresses the mRNA levels of PRL and IGFBP1 of HEnSCs by not only downregulating Forkhead box O1 (FOXO1) expression but also directly decreasing the histone H3K27 acetylation levels of the promoter regions of PRL and IGFBP1 (35). Of interest, as the only hormone that lowers blood glucose in human body, insulin has also been found to associate with decidualization. Decidualization is a highly energy-dependent process requiring adequate glucose uptake. Insulin decreased both mRNA and protein levels of GLUT1 in a dose-dependent manner in decidualizing HEnSCs, inhibiting glucose uptake in high insulin concentrations, which may have a negative impact on decidualization by reducing energy production (36). A high insulin pregnant mouse model showed a lower level of serum estrogen (E2), P4, FSH and LH on Gd 6-8 and an impaired function of endometrial angiogenesis compared to those in the control, suggesting that hyperinsulinemia may impede decidualization by disrupting reproductive hormones and restraining SA remodeling (37). These observations indicate that normal level of glucose is indispensable for decidualization. In addition, uterus can store glucose as glycogen. Glycogen level decreases in mouse uterine epithelium before implantation, but dramatically increases in stroma during decidualization. Although the role of glycogen in the decidua is unclear, it appears to be vital in sustaining the decidualization process (38).

Decidualization is accompanied by altered expression of enzymes involved in carbohydrate metabolism. In human, the level phosphofructokinase 1 is higher while fructose bisphosphatase 1 is lower in DSCs compared with EnSCs, leading to the accumulation of fructose-1,6-bisphosphate (FBP) in DSCs. This process can be promoted by E2 plus P4, or the supernatant of primary trophoblast cells. In a spontaneous abortion-prone mouse model, intraperitoneal injection of FBP increased FBP levels in both uterus and plasma and induced a high percentage of cyclooxygenase-2 (COX-2) ^+^ M2-like macrophages, regulatory T cells (Tregs), and T helper 2 (Th2) cells, which improved decidualization and trophoblast invasion (39).

During decidualization, glycolysis-related genes, such as HK2 (converting glucose to G6P), G6PDH (catalyzing G6P for pentose phosphate pathway (PPP)), LDH (producing lactate), and PDK1 (repressing pyruvate dehydrogenase (PDH) by phosphorylating PDHE1) at implantation sites are significantly increased, accompanied with hypoxia-inducible factor 1α, c-MYC and PI3K-AKT signaling activation in mice at Gd 5, suggesting an activated Warburg-like glycolysis effect but suppressed oxidative metabolism in decidua during early pregnancy (40, 41). Moreover, an enhanced lactate communication mediated by MCT4 (exporting lactate) and MCT1 (importing lactate) promotes the proliferation of the undifferentiated cells in mice by providing acidic extracellular milieu (42). In contrast, Frolova’s team reported that inhibiting glycolysis by a glyceraldehyde-3-phosphate dehydrogenase inhibitor did not affect decidualization process of human and mouse EnSCs *in vitro (* 43). The ambiguous conclusion may be due to compensatory effects of additional energy metabolic pathways. However, blockade of G6PDH, which guided the rate-limiting step in the PPP, led to lower expression of decidualization markers in both human and mouse EnSCs and impaired decidualization in mice (32, 43, 44). Complete endometrial repair is one of the premises of stromal cell differentiation and normal decidualization (45). Inhibiting glycolysis during endometrial repair not only reduces the hypoxia signaling and endometrial cell proliferation, but also disrupts inflammatory response in mouse model. This reflects the significance of glycolysis for decidualization in another way (46).

### 2.2 Lipid metabolism and DSC differentiation

In addition to carbohydrate, lipid also acts as an important source of energy for metabolism and homeostasis. Lipid distributes regularly in the peri-implantation uterus so as to supply raw material and energy for endometrial-decidual transformation (47). An early study reported that saturated fatty acids increased (43% to 64%) after induction of decidualization by Concanavalin A in pseudo-pregnant murine uterus. Meanwhile, polyunsaturated fatty acids (PUFAs) (15% to 10%) and sterols (19% to 4%) were decreased (48). Lipid metabolic regulation is implicated in the development of decidualization process. High-fat/high-sugar-exposed mice had much lighter deciduomas and obviously lower level of PRL-related protein than those intaking standard mouse chow. Similarly, HEnSCs cultured with palmitic acid had remarkably decreased level of PRL and IGFBP1. Furthermore, levels of autophagy regulators acetyl CoA carboxylase and ULK1 were higher in stimulated mouse uterine horns than unstimulated horns, indicating an induction of autophagy during decidualization. While autophagy was impaired by excessive fatty acid exposure in EnSCs of both mice and humans. Diet-induced obesity may impair EnSC decidualization partly *via* impaired autophagy (49). Fatty acids and the β-oxidation pathway also play a significant role in oocyte and embryo development (50, 51). Carnitine palmitoyltransferase-1 (CPT1) serves as a rate-limiting enzyme in fatty acid β-oxidation pathway to transfer acyl-CoA into the inner mitochondrial membrane, and its knocking down and adding β-oxidation inhibitor Ranolazine can impair decidualization of HEnSCs. It was investigated in HEnSCs that the expression levels of CPTA1 and CPT2 were upregulated in decidualizing cells compared to non-decidualizing cells (29). In mice, CPTA1 knockdown impaired decidualization with a significant reduced level of cell proliferation markers (proliferating cell nuclear antigen and cyclinD3) (52). Pup number is also markedly reduced under simultaneous inhibition of the β-oxidation pathway and PPP, while it can be recovered after the end of treatment period (53). Acting as lipid mediators, PGs play an essential role in embryo implantation, maternal-fetal interface construction and labor initiation (54). Interestingly, they can also act as signaling molecules to regulate decidualization. In human, 15-hydroxyprostaglandin dehydrogenase (15-PGDH) is a degrading enzyme of PG. PG transporter (PGT), a candidate molecule of PG carriers, can help transport PG into cells. Inhibiting 15-PGDH promotes a shift to a mesenchymal pattern of trophoblasts and DSCs depending on the PGT-mediated transport of PGE2, further promoting trophoblast differentiation and decidualization (8). Additionally, endothelial-derived prostacyclin and PGE2 can accelerate decidualization by increasing intercellular cAMP in the perivascular stroma *via* a paracrine mechanism, which can be induced by hemodynamic forces. The enhanced PG-driven decidualization could also be mediated by COX-2, PGE2 and prostacyclin (55). A recent single-cell analysis study of human decidual cells also indicated that PGE2-mediated decidualization depends upon PG-dependent induction of PGE2 receptor 2 and protein kinase A (PKA) (56).

Metabolism of phospholipids is another important part of lipid metabolism in cancer and pregnancy (57). Two lipid phosphate phosphatases (LPP1 and LPP3), lysophosphatidic acid (LPA) receptor LPAR1, sphingosine-1-phosphate (S1P) receptor 3, and S1P phosphatase are highly expressed in decidualized HEnSCs (10, 58). Decidualization was deficient in sphingosine kinase-1^–/–^ mice, suggesting that LPA and S1P receptors, as well as their metabolizing enzymes play important roles in decidualization (59–61). In mice, autotaxin-LPA-LPA3 signaling in embryo-epithelial interaction area induces decidualization *via* heparin-binding epidermal growth factor (HB-EGF)/COX-2-Bmp2/Wnt4 signaling pathways (62). Consistently, Bmp2/Wnt4 signaling takes part in decidualization and acts as downstream of HB-EGF signaling (63, 64). Sphingolipids also participate in pregnancy. Serine palmitoyl-transferase (SPT) is the first key enzyme of *de novo* sphingolipid synthesis pathway. It is composed of three major subunits (Sptlc1, Sptlc2, and Sptlc3) and two small subunits (Ssspta and Sssptb) (65, 66). During decidualization, the mRNA levels of three subunits of the SPT holoenzyme (Sptlc1, Sptlc2, and Ssspta) were significantly upregulated in mouse uterine stromal cells. Blocking the *de novo* sphingolipid synthesis with a specific inhibitor of SPT impeded the decidualization, suggesting that sphingolipid was an essential lipid compound in decidualization (67).

### 2.3 Amino acid metabolism and DSC differentiation

Amino acids like arginine and leucine have influenced on the embryo implantation and fetal growth in both humans and rodents (68, 69). Arginine addition during early pregnancy induces embryo implantation through activating the PI3K/PKB/mTOR/NO signaling pathway in rats (69). cAMP can induce argininosuccinate synthase (Ass1) through PKA/phosphorylated cAMP-response element binding protein signaling pathway to supply ample L-arginine for mouse decidualization. While L-arginine at high concentration downregulates Ass1 and argininosuccinate lyase expression to maintain the homeostasis of L-arginine (70). Tryptophan is also an essential amino acid for pregnancy. Nearly 95% tryptophan enters kynurenine pathway, during which indoleamine 2,3-dioxygenase (IDO) and tryptophan 2,3-dioxygenase are the rate-limiting enzymes (71). Tryptophan (enters cells *via* solute carrier family 7, member 5) and its metabolite kynurenine enhance HEnSC decidualization *via* aryl hydrocarbon receptor (AHR) pathway, which is significantly stimulated by gamma interferon (IFN-γ) (72, 73). Moreover, HCG regulated interleukin (IL) 4-induced gene 1 (IL4I1) expression and secretion from human endometrial epithelial cells through polyamine metabolism. Both IL4I1-catalyzed indole-3-pyruvic acid and its metabolite indole-3-aldehyde from tryptophan were able to induce *in vitro* decidualization of HEnSC *via* AHR-epiregulin pathway (74). Recently, Tang et al. delineated an activated glutamine (Gln) metabolism in decidualization. They uncovered the fundamental support of Gln-glutamic acid (Glu)-α-ketoglutarate (α-KG) metabolism flux for successful decidualization through Gln-Glu-αKG-dependent H3K27 demethylation in promotor regions of PRL and IGFBP1, while patients with RSA exhibited impaired decidualization owing to deficient Gln metabolism (75).

**Figure 1:**
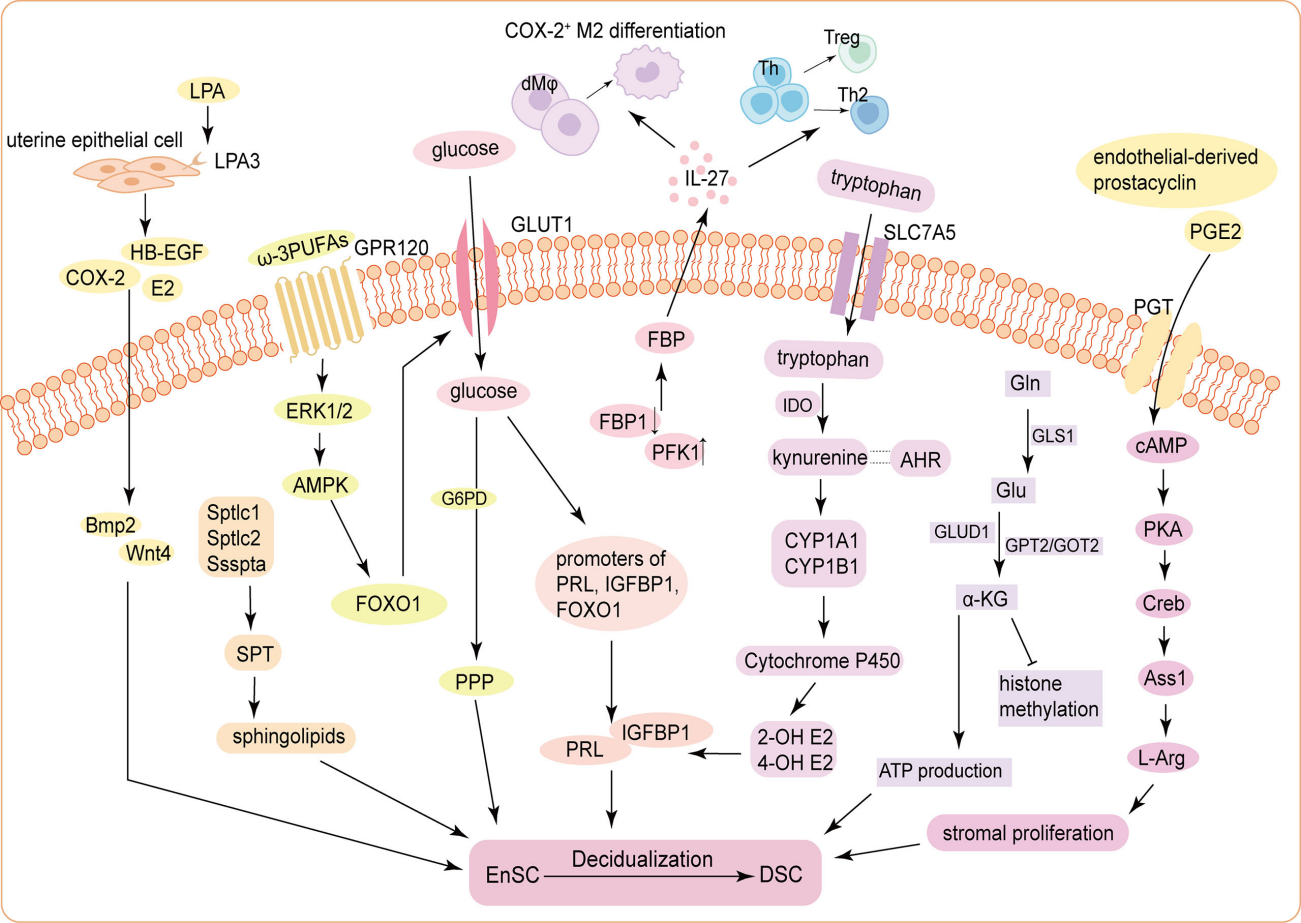
Schematic mechanism of the energy metabolism during decidualization. (1) ATX-LPA-LPA3 signaling in the uterine epithelium induces decidualization *via* HB-EGF/COX-2-Bmp2/Wnt4 signaling pathways. Three subunits of the SPT holoenzyme (Sptlc1, Sptlc2, and Ssspta) are significantly up-regulated in mouse USCs, resulting in *de novo* synthesis of sphingolipids, to promote decidualization. (2) Glucose uptake is increased and is indispensable for decidualization by activating the histone modification status of the promoters of PRL, IGFBP1 and FOXO1. The FBP level in DSCs is significantly increased, which elevates IL-27 expression, triggering COX-2^+^ M2-like macrophages differentiation, Treg expansion, and Th2 bias to improve decidualization and trophoblast invasion. (3) cAMP enhances stromal cells proliferation through PKA/p-Creb/Ass1/L-Arg signaling pathway. Tryptophan enters into cells *via* SLC7A5 and stimulates the expression of PRL and IGFBP1 through kynurenine pathway. Accumulated α-KG derived from activated glutaminolysis contributes to ATP production and decidualization (4) The influences of carbohydrate metabolism, lipid metabolism and amino acid metabolism on DSC differentiation are not independent. For example, GPR120, receptor of ω-3 PUFAs, functions to promote decidualization by upregulating GLUT1-mediated glucose uptake and G6PD-mediated PPP of HEnSCs in a ERK1/2 and AMPK pathway. Endothelial-derived prostacyclin and PGE2 can accelerate decidualization by increasing intercellular cAMP in the endometrial perivascular stroma. ATX, autotaxin; LPA, lysophosphatidic acid; HB-EGF, heparin-binding epidermal growth factor; COX-2, cyclooxygenase-2; Bmp2, bone morphogenetic protein 2; SPT, serine palmitoyltransferase; USCs, uterine stromal cells; E2, estrogen; PRL, prolactin; IGFBP1, insulin growth factor binding protein 1; FOXO1, Forkhead box O1; FBP, fructose-1;6-bisphosphate; DSCs, decidual stromal cells; PFK1, phosphofructokinase 1; FBP1, fructose-bisphosphatase 1; Th, helper T cell; Treg, regulatory T cell; cAMP, cyclic adenosine monophosphate; PKA, protein kinase A; p-Creb, phosphorylated cAMP-response element binding protein; Ass1, Argininosuccinate synthase; Asl, Argininosuccinate lyase; L-Arg, L-Arginine; SLC7A5, solute carrier family 7; member 5; IDO, indoleamine 2;3-dioxygenase; AHR, Aryl hydrocarbon receptor; CYP1A1, Cytochrome P450 1A1; CYP1B1, Cytochrome P450 1B1; 2-OH E2, 2-hydroxy estradiol; 4-OH E2, 4-hydroxy estradiol; α-KG, α-ketoglutarate; Gln, glutamine; GLS1, glutaminase 1; Glu, Gln-glutamic acid; GLUD1, Glu dehydrogenase 1; GPT2, glutamic-pyruvic transaminase 2; GOT2, glutamic-oxaloacetic transaminase 2; GPR120, G-protein-coupled receptor 120; PUFA, Polyunsaturated fatty acids; GLUT1, glucose transporter-1; G6PD, glucose-6-phophate dehydrogenase; PPP, pentose-phosphate pathway; HEnSCs, human endometrial stromal cells; ERK1/2, extracellular regulated protein kinases; AMPK, adenosine 5’-monophosphate (AMP)-activated protein kinase; PGE2, prostaglandin E2; PGT, prostaglandin transporter.

The influences of carbohydrate metabolism, lipid metabolism and amino acid metabolism during decidualization are not independent (**Figure 1**). Intake of ω-3 PUFAs has positive effects on pregnancy outcome (76). G-protein-coupled receptor 120, the receptor of ω-3 PUFAs, can facilitate decidualization by upregulating GLUT1-mediated glucose uptake and G6PD-mediated PPP of HEnSCs (77). In obese pregnant mice, decidualization of endometrium is compromised with significant decrease in key glycolytic enzyme (hexokinase2, pyruvate kinase M2 (PKM2) and LDHA) levels, indicating that disorders of lipid metabolism may impair glycolysis of EnSCs and decidualization (78). Additionally, blocked decidualization of HEnSCs by β-oxidation inhibitor happened to resume after several days due to a compensatory up-regulation of GLUT1 level and an enhancement in glucose metabolism, suggesting a delicate relationship between glucose and lipid metabolism during decidualization (53). Such phenomena obviously exist, but our discoveries concerning it are still limited.

Nevertheless, the energy metabolic profile of decidualization still has many undiscovered fields to be explored. Notably, substances involved in energy metabolism also mediate the communication between DICs and DSCs to favor decidualization. For example, Daniele Croxatto et al. found that both IDO and PGE2 play important roles in DSC-mediated inhibition of natural killer (NK) cell and dendritic cell (DC) function (79). Next, we will focus on DICs and further elaborate possible mechanisms of the interplay among DICs, DSCs and trophoblasts during decidualization.

## 3 DICs during decidualization

During decidualization, unique immune cell niches are found in decidual bed, suggesting immune cells are involved in decidualization process. A large number of immune cells are recruited to the maternal-fetal interface and then called DICs. DICs, mainly including NK cells, T cells, macrophages and DCs, account for 30%–40% of the total decidual cells in early pregnant uterus. Great importance in immune tolerance has been attached to DICs, while the roles of DICs in decidualization are nonnegligible.

### 3.1 NK cells and decidualization

NK cells increase dramatically during the secretory menstrual phase and early pregnancy, constituting approximately 70% of DICs. Unlike 90% of peripheral blood NK cells with a CD56^dim^CD16^+^phenotype, most decidual NK cells (dNKs) have a CD56^bright^CD16^-^ phenotype with reduced cytotoxicity. CD56^bright^CD16^-^ NK cells are capable of producing various soluble factors, such as vascular endothelial growth factor (VEGF), placental growth factor (PLGF), IL-8, IL-1β, CCL2/monocyte chemoattractant protein 1 (80). NK cells play an important role in trophoblast invasion and SA remodeling. Women with antiphospholipid antibodies circulation have much lower absolute levels of NK cells than healthy women and have a higher risk of suffering from reproductive failures such as miscarriage, preeclampsia, infertility and failure of IVF, which implies that the lack of NK cells may be associated with insufficient preparation of decidualized endometrium for embryo invasion (81). A single-cell transcriptomics atlas of the maternal-fetal interface between 6-14 weeks of gestation identified three main dNK subsets with co-expression of CD49A and CD9: dNK1, characterized by CD39, CYP26A1 and B4GALNT1; dNK2, defined by ANXA1 and ITGB2; and dNK3, with expression of ITGB2, CD160, KLRB1 and CD103, but not CD127, among which dNK1 subset can be primed metabolically through increased expression of glycolytic enzymes, which is a crucial part of carbohydrate metabolism during decidualization as mentioned above (6).

The phenotype and function of uterine NK cells (uNKs) are regulated by a variety of cytokines, among which IL-15 plays a key role in function reprogramming of uNKs (82). IL-15 is expressed at all stages of human menstrual cycle and in first trimester decidua, the level of which can be up-regulated by both PGE2 and IFN-γ in cultures of HEnSCs (83). Whereas transcription of IL-15 initiates following the onset of decidualization and terminates on Gd11 in mouse uterus (84). uNKs responding to IL-15 can upregulate the expression of VEGF and PLGF, both related to potent pro-angiogenic effects *in vitro* and *in vivo (* 82). Upon induction with IL-15, the proliferation, cytolytic capacity and IFN-γ production were also severely impaired in NK cells isolated from peripheral blood of healthy donors (79). Compared to wild type mice, IL-15 deficiency mice had neither uNKs nor SA remodeling at the blastocyst implantation sites, and lacked integrated decidualization. The fact is that uNKs upregulate IL-15 and IL-15Rα in stromal fibroblast for decidualization promoting, which in turn provides a niche for uNK proliferation and recruitment, mirroring the essential role of IL-15-responding uNKs during decidualization (85). uNKs also actively and systematically eliminate senescent EnSCs only upon decidualization, which remodels and rejuvenates the endometrium for embryo implantation (86). It was also showed that the isolated primary dNKs secreted IL-25 time dependently, the level of which was apparently increased following co-culture with HEnSCs, further promoting the decidualization *in vitro (* 87). This example may provide another perspective that dNKs can facilitate decidualization by interacting with EnSCs.

### 3.2 DCs and decidualization

Despite the magnitude of DCs in decidua is few (only account for 1% of the DICs), DCs are the most prominent antigen presenting cells for immune tolerance during pregnancy, especially CD11c^+^ DCs (88). The number of DCs reached a maximum in mouse uterus at Gd 5.5. This early accumulation has been connected to the transient inflammatory milieu of WOI (89). Loss of DC alone directly leads to obstacle in decidualization and embryo implantation. Moreover, DC depletion also impairs NK cell differentiation and DSC proliferation, resulting in a reduced breeding efficiency in mice (90). The above two points indicate the irreplaceable function of DCs in addition to the classical immunomodulatory effects during decidualization.

### 3.3 Macrophages and decidualization

Macrophages represent 10%-20% of the leukocytes at the maternal-fetal interface (91). Macrophages are classified into two groups: microbicidal and pro-inflammatory M1 or classically activated macrophages, and anti-inflammatory M2 or alternatively activated macrophages (92, 93). Macrophages are distributed throughout mouse uteri before implantation, but they are dispelled in the decidual zone after implantation, this variation may protect developing blastocyst from inflammatory injury by macrophages (94). Decidualization inducer MPA can drive a M2 differentiation in human monocytic cell line THP-1. The MPA-stimulated M2 promote the decidualization of HEnSCs and the invasion of trophoblasts (95). Macrophages also play a critical role in supporting the extensive vascular network in corpus luteum and production of P4. In macrophage-depleted mice, ovaries were hemorrhagic with a highly irregular architecture and extensive structural disruption in most corpora luteal. Meanwhile, plasma P4 was reduced significantly. Adverse pregnancy outcomes can be rescued by exogenous P4 or bone marrow-derived CD11b^+^F4/80^+^ monocytes/macrophages, suggesting that macrophages may promote decidualization by maintaining normal structure of corpus luteum and P4 levels (96). Decidual macrophages have higher level of IL-27 receptor (IL-27R) than other decidual cells. The co-culture of decidual macrophages and HEnSCs, especially IL-27^over^ HEnSCs, induces COX-2^+^M2-like macrophage differentiation. In turn, COX-2^+^ decidual macrophages promote decidualization and trophoblast invasion, thus preventing pregnancy loss induced by the absence of the IL-27/IL-27RA signal axis (39).

### 3.4 T cells and decidualization

CD3^+^ T cells appear to be relatively sparse in the human decidua, accounting for 10%–20% of DICs. 30%–45% CD3^+^ T cells are CD4^+^ T cells and 45%–75% are CD8^+^ T cells (97). Developmental process of decidualization reduced the T cell chemo-attractants production under inflammatory conditions due to a gene-specific intrinsic inability to recruit T cells from the blood independent of inflammatory signaling (98). In women with antiphospholipid syndrome, prominently reduced absolute level of T cells was found, which may be associated with insufficient decidualization of endometrium for embryo invasion (81). Pregnancy is recognized as a Th2-like predominant immunity event (99–101), characterized by increased Th2-type cytokines (IL-4, IL-10, IL-5 and IL-13) and decreased Th1-type cytokines (tumor necrosis factor (TNF)-α, TNF-β, INF-γ and IL-2). IGFBP7 functions as a decidualization modulator in EnSCs. Inhibition of IGFBP7 shifts mouse uterine cytokines to Th1-type dominance and represses decidualization, resulting in pregnancy failure (102). This may indicate that Th2 bias existing at the maternal-fetal interface is essential for decidualization.

Now the Th1/Th2 paradigm has been expanded into Th1/Th2/Th17 and Treg paradigm (103, 104). Th1, Th2, and Th17 cells are defined by their cytokine production (IFN-γ, IL-4, and IL-17, respectively). Tregs are defined by Foxp3 expression. They have different but partly overlapping chemokine receptor profiles (105). Tregs are significantly enriched in decidua and display a more homogenous suppressive phenotype with more frequent expression of Foxp3, human leukocyte antigen DR (HLA-DR), and cytotoxic T-lymphocyte antigen 4 (CTLA-4) (104). However, the underlying mechanisms of how do Tregs and Th17 cells function during decidualization is not clear to date, which needs further investigation.

### 3.5 The crosstalk of DSCs, trophoblasts and DICs during decidualization

Successful pregnancy is a very complex biological regulation process, which not only requires the mother to tolerate the fetus as an allograft, but also relies on well-developed placenta and decidua. A complex and reciprocal interaction between the mother and fetus is required to maintain the growth and development of the fetus in the uterus until delivery. For example, DICs attempt to attack allogeneic fetal placental trophoblasts by producing the Th1 type cytokine TNF-α, while trophoblasts intend to defend themselves by producing the Th2-type cytokines, in which situation, DSCs play an intermediary role in reconciling the relationship between DICs and trophoblasts and creating a harmonious Th2 bias microenvironment.

**Figure 2:**
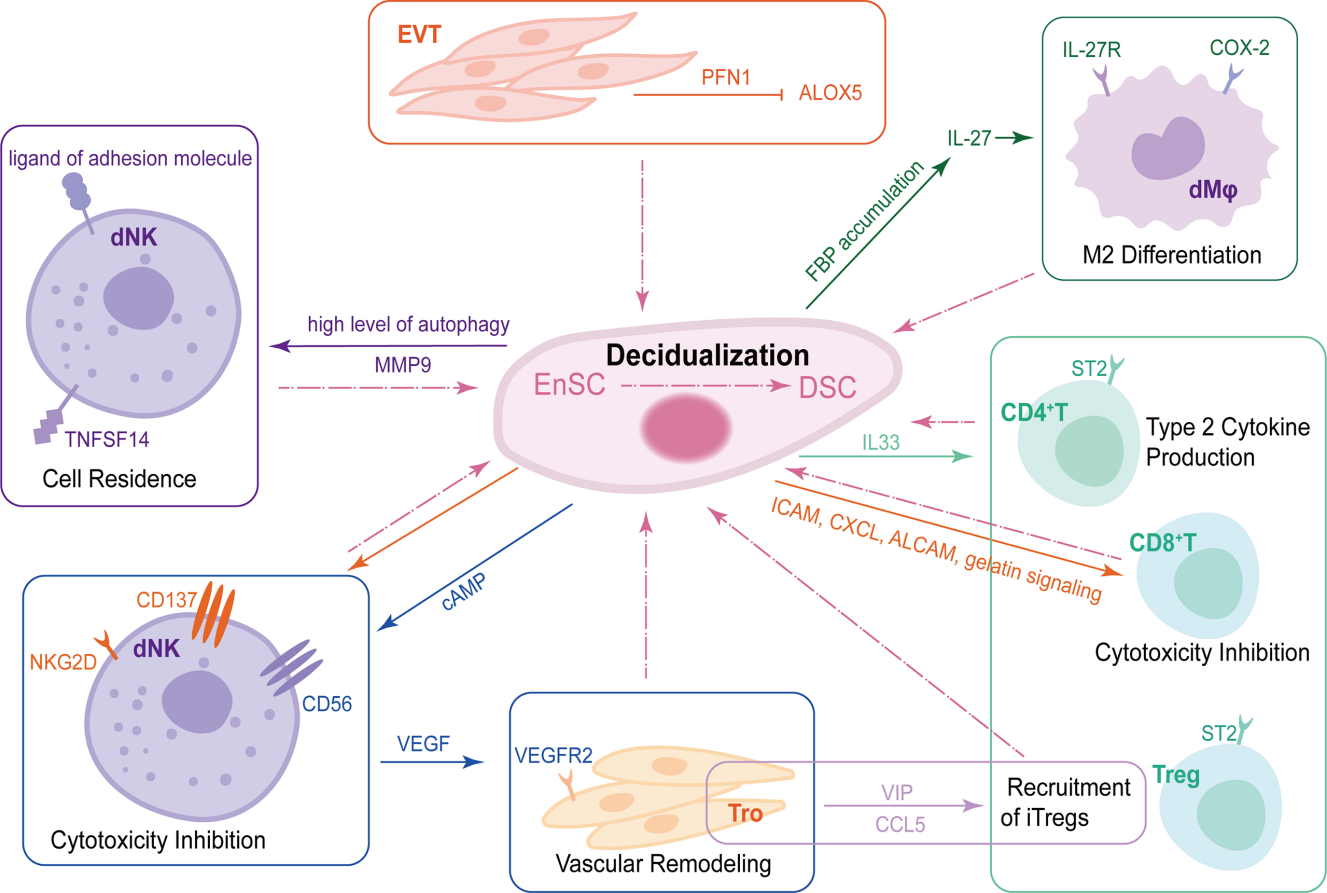
The crosstalk of DSCs, trophoblasts and DICs during decidualization. During decidualization, high level of autophagy in DSCs upregulates TNFRSF14. The TNFSF14-TNFRSF14 signal contributes to the increased adhesion ability of DSCs by upregulating MMP9 expression, further facilitating the residence of dNKs in decidua. Moreover, DSCs increase the expression of CD56 on NK cells by producing abundant cAMP. CD56^bright^ dNKs can promote vascular remodeling of trophoblast *via* VEGF-VEGFR2 interaction. DSCs also produce IL-33 to regulate decidualization by interacting with ST2^+^ DICs and promoting type 2 responses. EnSCs mediate maternal immune tolerance *via* ICAM, CXCL, ALCAM and gelatin signaling on CD8^+^T cells as well as NKG2D and CD137 on uNKs to inhibit their cytotoxicity. In addition to DSCs, EVT-secreted PFN1 promotes stromal cell decidualization *via* the down-regulation of ALOX5. Trophoblasts mediate the recruitment of iTregs, one of the important components of DICs, in a VIP/CCL5 pathway. Trophoblasts also promote the enrichment of FBP in DSCs, which can upregulate IL-27, further maintaining normal pregnancy by inducing decidual COX-2^+^ M2 macrophage differentiation. DSCs, decidual stromal cells; DICs, decidual immune cells; TNFSFR14, tumor necrosis factor (TNF) superfamily member receptor 14; TNFSF14, TNF superfamily member 14; MMP9, matrix metalloproteinase 9; dNKs, decidual natural killer cells; cAMP, cyclic adenosine monophosphate VEGF, vascular endothelial growth factor; VEGFR2, vascular endothelial growth factor receptor2; ICAM, intracellular adhesion molecule 1; CXCL, chemokine C-X-C motif ligand; ALCAM, activated leukocyte cell adhesion molecule; EVT, extracellular villus trophoblast; PFN1, profilin 1; ALOX5, lipoxygenase arachidonate 5-lipoxygenase.

Maternal immune cells can be educated by embryonic trophoblasts to develop a unique phenotype and maintain fetal tolerance. Trophoblasts-derived thymic lymphopoietin (TSLP) contributes to instructing DCs to secrete IL-10 and CCL17, so as to induce Th2 bias at the maternal-fetal interface. TSLP-activated decidual DCs induce proliferation and differentiation of CD4^+^ CD25^+^Foxp3^+^ Tregs through transforming growth factor beta 1 (TGF-β1). Then, decidual Tregs can promote invasiveness and HLA-G expression of trophoblasts, leading to preferential production of Th2 cytokines and reduced cytotoxicity in decidual CD56^bright^CD16^-^ NK cells (106). Trophoblasts also contribute to the increased expression of T cell immunoglobulin and mucin domain-containing protein 3 (Tim-3), programmed cell death protein 1 and CTLA-4 on DICs to further develop a regulatory phenotype for fetal tolerance in an HLA-C-restricted manner (107–111). Tim-3/CTLA-4 pathways, in turn, might operate within the functional immune-modulatory network not only to promote maternal-fetal tolerance but also to improve trophoblast function through DIC-trophoblast interaction dependent on IL-4 and IL-10 (112). DSCs provide fundamental decidual bed for the recruitment, differentiation and maturation of DICs. DSC-derived CCL2 enhances proliferation and inhibits apoptosis of DICs. CCL2 induces Th2 bias by upregulating Th2 type transcription factor GATA-3 and downregulating Th1 type transcription factor T-bet. Meanwhile, Th2 cytokines IL-4 and IL-10 in turn increase CCL2 production by DSCs (113), which forms a positive regulatory loop. Additionally, CCL2 secreted by DSC inhibits NK cells cytotoxicity by upregulating suppressors of cytokine signaling 3 (114). The crosstalk of DSCs, trophoblasts and DICs also exists during decidualization (**Figure 2**).

Cumulative evidence has suggested that stromal cells can modulate immune cell phenotype and maintain normal functions of immune cells in many ways, which in turn contribute to the decidualization of EnSCs. The crosstalk between DSCs and DICs is important for decidualization and pregnancy maintenance. In human, differentiation from decidual CD34^+^ hematopoietic precursors to mature CD56^bright^CD16^−^KIR^+/−^ NK cells can be obtained upon co-culture with DSCs in absence of added cytokines (115). DSCs severely impaired cytolytic activity and IFN-γ production of dNKs (79). High level of autophagy in DSCs facilitates the adhesion and retention of dNKs, further promoting decidualization. Patients with unexplained spontaneous abortion may display insufficient DSC autophagy and dNK residence, which can be prevented by rapamycin, an autophagy inducer (116). A single−cell RNA sequencing study found a unique insulin-like growth factor 1 (IGF1) ^+^ stromal cell that may participate in the initiation of decidualization. Meanwhile, amphiregulin (AREG)^+^ NK cells could accelerate decidualization and extra-villous trophoblast (EVT) invasion by interacting with IGF1^+^ stromal cells *via* AREG−IGF1 and AREG−CSF1 regulatory axe (117). cAMP is a critical substance for efficient decidualization (118). Decidualization-derived cAMP can not only promote FOXO1-mediated CD56 upregulation in NK cells, but also promote VEGF production and enhance vascular remodeling ability of NK cells during the critical period of decidualization (119). TNF receptor 1 (TNFR1) expression is significantly upregulated among DSCs and macrophages in RSA decidua, which mediates TNF-α-induced excessive stromal senescence following decidualization through TNFR1/p53/p16 pathway (120). DSC-derived IL-33 increases Th2 cytokine (IL-4, IL-13, and IL-10) with a concomitant decrease in TNF-α of dNK cells through NF-κB signaling (121). Daniele Croxatto et al. also found that DSCs mediated the inhibition of DC differentiation and its function to induce allogeneic T cell proliferation (79).

In mice, there is a subtle interaction between IL-33-producing non-immune cells (myometrial fibroblasts, decidual endothelial and stromal cells) and ST2^+^ immune cells during decidualization. IL-33 signaling promotes decidualization and vascularization in early pregnancy, which in turn lays the foundation for optimal outcomes at later stages of pregnancy (122). Jiang’s team first reported the role of non-decidualized EnSCs on embryo implantation in CD-1 mouse model at the post-implantation stage by single-cell RNA-sequencing. The results showed that EnSCs exhibited the most outgoing signals, whereas CD8^+^T cells received most of the incoming signals at Gd 4.5 and 5.5. EnSCs actively mediate maternal immune tolerance *via* intracellular adhesion molecule 1, CXCL, activated leukocyte cell adhesion molecule and gelatin signaling on CD8^+^T cells as well as NKG2D and CD137 on uNKs to inhibit their cytotoxicity (123). Taken together, the interplay between stromal cells and immune cells is tightly correlated with decidualization.

Trophoblasts are also critical in the complex interaction of human decidualization. Trophoblasts, DSCs and sex hormones jointly contribute to the high level of IDO, a tryptophan-catabolizing enzyme, in macrophages during early pregnancy, which triggers M2 polarization, promotes invasion-related molecules (CXCL12 and Bmp2) expression and trophoblast proliferation (124). Vasoactive intestinal peptide, a pleiotropic peptide produced by trophoblasts, might participate in the decidualization process by inducing differentiation markers and increasing induced Tregs (iTregs) chemokine CCL5 production (125, 126). In first trimester decidua, CD68^+^macrophages and DSCs express arachidonate 5-lipoxygenase (ALOX5), a lipoxygenase enzyme involved in the synthesis of proinflammatory leukotrienes. EVT-derived proflin1 can promote stromal cell decidualization *via* suppressing ALOX5 in human monocyte cell line and primary HEnSCs. These showed a key role of HEnSC-EVT crosstalk during decidualization (127).

Trophoblasts also promote the accumulation of FBP in DSCs during decidualization. FBP increases IL-27 expression in DSCs in a PKM2/ERK1/2/c-FOS-dependent manner. On the one hand, IL-27 triggers decidual COX-2^+^ M2-like macrophage differentiation (39), on the other hand, IL-27 facilitates decidualization by increasing the expression of E2 and P4 receptors *via* STAT3 phosphorylation (128). CD44 and its isoform CD44v3 are transmembrane glycoproteins expressed in EnSCs. CD44v3-knockdown led to downregulation of PRL and IGFBP1, suppression of HEnSC proliferation and decidualization, and inhibition of trophoblast outgrowth (129). Additionally, abnormal elevation of IGF2BP3 (insulin-like growth factor 2 mRNA-binding protein 3) in HEnSCs impaired decidualization by inhibiting TGF-β1 pathway, disrupting maternal-fetal cytokine crosstalk and suppressing trophoblast invasion (130). In mice, EnSC-trophoblast crosstalk can also be mediated by EVs. EnSCs secrete EVs in HIF2α-Rab27b pathway during decidualization. Matrix metalloproteinase-2, a prominent EV cargo protein, is a pivotal mediator contributing to uterine decidualization and angiogenesis programs (131). Collectively, healthy decidualization requires moderate and effective dialogue between trophoblasts and decidual cells.

## 4 Conclusion and prospects

Decidualization is a significant biological event including intricate mechanisms. However, much less attention has been given to decidua than trophoblasts. Ultimately, decidualization acts as the “soil” for the “seed” (embryo implantation). EnSCs of recurrent abortion patients show an aberrant response to decidualization *in vitro*, manifested by attenuated PRL production and prolonged and enhanced prokineticin 1 level. Some hypotheses also suggest the endometrial selectivity of embryo by decidualized EnSCs. For which, the inclination of miscarriage may be due to the failure of embryo selection by abnormal decidualization (22, 132). A strong signature of dysregulated decidual gene expression (e.g., decreased levels of IGFBP1, glycodelin, PRL and IL-15) also emerged among women with severe preeclampsia compared with those experienced normal pregnancies (133, 134).

In this review, we elaborate the crosstalk of functional cells at the maternal-fetal interface during the decidualization process, with a special focus on the metabolic profiles on the initiation and maintenance of decidualization. Integrated differentiation of DSCs as well as DICs and trophoblasts are significant components of normal decidualization and subsequent healthy pregnancy. Dysfunction of each part will result in pathological pregnancy. Our understanding of how the trophoblasts, DSCs and DICs network orchestrates crucial events during normal pregnancy and pregnancy complications remains incomplete. Clues have also been found on epigenetic regulation to immune cells together with metabolism alteration, indicating there are abstruse association among immune, metabolic and epigenetic approach accompanied by differentiation of DSCs in the process of decidualization. It will give us more hints in the future research in order to better understand decidualization.

## Author contributions

XM and CC drafted the manuscript. JQ coordinated the literature search and figure preparation. SW and LC revised the manuscript. All authors reviewed the manuscript.

## References

[1] Lim HJ Wang H. Uterine disorders and pregnancy complications: insights from mouse models. J Clin Invest (2010) 120(4):1004–15. doi: 10.1172/jci41210 PMC2846054 20364098

[2] Ramathal CY Bagchi IC Taylor RN Bagchi MK. Endometrial decidualization: of mice and men. Semin Reprod Med (2010) 28(1):17–26. doi: 10.1055/s-0029-1242989 20104425 PMC3095443

[3] Cha J Sun X Dey SK. Mechanisms of implantation: strategies for successful pregnancy. Nat Med (2012) 18(12):1754–67. doi: 10.1038/nm.3012 PMC6322836 23223073

[4] Kane NM Jones M Brosens JJ Kelly RW Saunders PT Critchley HO. TGFβ1 attenuates expression of prolactin and IGFBP-1 in decidualized endometrial stromal cells by both SMAD-dependent and SMAD-independent pathways. PLoS One (2010) 5(9):e12970. doi: 10.1371/journal.pone.0012970 20885978 PMC2945765

[5] Ng SW Norwitz GA Pavlicev M Tilburgs T Simón C Norwitz ER. Endometrial decidualization: the primary driver of pregnancy health. Int J Mol Sci (2020) 21(11):4092. doi: 10.3390/ijms21114092 32521725 PMC7312091

[6] Vento-Tormo R Efremova M Botting RA Turco MY Vento-Tormo M Meyer KB. Single-cell reconstruction of the early maternal-fetal interface in humans. Nature (2018) 563(7731):347–53. doi: 10.1038/s41586-018-0698-6 PMC7612850 30429548

[7] Maruyama T Yoshimura Y. Molecular and cellular mechanisms for differentiation and regeneration of the uterine endometrium. Endocr J (2008) 55(5):795–810. doi: 10.1507/endocrj.k08e-067 18580040

[8] Pang H Lei D Chen T Liu Y Fan C. The enzyme 15-hydroxyprostaglandin dehydrogenase inhibits a shift to the mesenchymal pattern of trophoblasts and decidual stromal cells accompanied by prostaglandin transporter in preeclampsia. Int J Mol Sci (2023) 24(6):5111. doi: 10.3390/ijms24065111 36982197 PMC10049104

[9] Wang W Vilella F Alama P Moreno I Mignardi M Isakova A. Single-cell transcriptomic atlas of the human endometrium during the menstrual cycle. Nat Med (2020) 26(10):1644–53. doi: 10.1038/s41591-020-1040-z 32929266

[10] Brünnert D Sztachelska M Bornkessel F Treder N Wolczynski S Goyal P. Lysophosphatidic acid and sphingosine 1-phosphate metabolic pathways and their receptors are differentially regulated during decidualization of human endometrial stromal cells. Mol Hum Reprod (2014) 20(10):1016–25. doi: 10.1093/molehr/gau051 24994816

[11] Huang JY Yu PH Li YC Kuo PL. NLRP7 contributes to *in vitro* decidualization of endometrial stromal cells. Reprod Biol Endocrinol (2017) 15(1):66. doi: 10.1186/s12958-017-0286-x 28810880 PMC5558772

[12] Jiang Y Kong S He B Wang B Wang H Lu J. Uterine Prx2 restrains decidual differentiation through inhibiting lipolysis in mice. Cell Tissue Res (2016) 365(2):403–14. doi: 10.1007/s00441-016-2383-0 26987819

[13] Murakami K Lee YH Lucas ES Chan YW Durairaj RP Takeda S. Decidualization induces a secretome switch in perivascular niche cells of the human endometrium. Endocrinology. (2014) 155(11):4542–53. doi: 10.1210/en.2014-1370 25116707

[14] Sroga JM Ma X Das SK. Developmental regulation of decidual cell polyploidy at the site of implantation. Front Biosci (Schol Ed) (2012) 4(4):1475–86. doi: 10.2741/s347 PMC4269479 22652887

[15] Pakrasi PL Jain AK. Cyclooxygenase-2 derived PGE2 and PGI2 play an important role *via* EP2 and PPARdelta receptors in early steps of oil induced decidualization in mice. Placenta (2008) 29(6):523–30. doi: 10.1016/j.placenta.2008.03.001 18407349

[16] Wang H Dey SK. Roadmap to embryo implantation: clues from mouse models. Nat Rev Genet (2006) 7(3):185–99. doi: 10.1038/nrg1808 16485018

[17] Yuan J Aikawa S Deng W Bartos A Walz G Grahammer F. Primary decidual zone formation requires scribble for pregnancy success in mice. Nat Commun (2019) 10(1):5425. doi: 10.1038/s41467-019-13489-4 31780662 PMC6882879

[18] Dey SK Lim H Das SK Reese J Paria BC Daikoku T. Molecular cues to implantation. Endocr Rev (2004) 25(3):341–73. doi: 10.1210/er.2003-0020 15180948

[19] Gellersen B Brosens JJ. Cyclic decidualization of the human endometrium in reproductive health and failure. Endocr Rev (2014) 35(6):851–905. doi: 10.1210/er.2014-1045 25141152

[20] Galliano D Bellver J. Female obesity: short- and long-term consequences on the offspring. Gynecol Endocrinol (2013) 29(7):626–31. doi: 10.3109/09513590.2013.777420 23514221

[21] Salker M Teklenburg G Molokhia M Lavery S Trew G Aojanepong T. Natural selection of human embryos: impaired decidualization of endometrium disables embryo-maternal interactions and causes recurrent pregnancy loss. PLoS One (2010) 5(4):e10287. doi: 10.1371/journal.pone.0010287 20422017 PMC2858209

[22] Teklenburg G Salker M Molokhia M Lavery S Trew G Aojanepong T. Natural selection of human embryos: decidualizing endometrial stromal cells serve as sensors of embryo quality upon implantation. PLoS One (2010) 5(4):e10258. doi: 10.1371/journal.pone.0010258 20422011 PMC2858159

[23] Conrad KP Rabaglino MB Post Uiterweer ED. Emerging role for dysregulated decidualization in the genesis of preeclampsia. Placenta (2017) 60:119–29. doi: 10.1016/j.placenta.2017.06.005 PMC5718949 28693893

[24] Sato S Solanas G Peixoto FO Bee L Symeonidi A Schmidt MS. Circadian reprogramming in the liver identifies metabolic pathways of aging. Cell (2017) 170(4):664–77.e11. doi: 10.1016/j.cell.2017.07.042 28802039 PMC7792549

[25] Harden SL Zhou J Gharanei S Diniz-da-Costa M Lucas ES Cui L. Exometabolomic analysis of decidualizing human endometrial stromal and perivascular cells. Front Cell Dev Biol (2021) 9:626619. doi: 10.3389/fcell.2021.626619 33585482 PMC7876294

[26] Kim ST Moley KH. Regulation of facilitative glucose transporters and AKT/MAPK/PRKAA signaling *via* estradiol and progesterone in the mouse uterine epithelium. Biol Reprod (2009) 81(1):188–98. doi: 10.1095/biolreprod.108.072629 PMC3093992 19208550

[27] Frolova A Flessner L Chi M Kim ST Foyouzi-Yousefi N Moley KH. Facilitative glucose transporter type 1 is differentially regulated by progesterone and estrogen in murine and human endometrial stromal cells. Endocrinology (2009) 150(3):1512–20. doi: 10.1210/en.2008-1081 PMC2654750 18948400

[28] Joost HG Bell GI Best JD Birnbaum MJ Charron MJ Chen YT. Nomenclature of the GLUT/SLC2A family of sugar/polyol transport facilitators. Am J Physiol Endocrinol Metab (2002) 282(4):E974–6. doi: 10.1152/ajpendo.00407.2001 11882521

[29] Citrinovitz ACM Hauke J Jauckus J Langhans CD Schwarz K Zorn M. Glucose and fatty acids catabolism during *in vitro* decidualization of human endometrial stromal cells. J Assist Reprod Genet (2022) 39(12):2689–97. doi: 10.1007/s10815-022-02637-3 PMC9790837 36308613

[30] Yang M Li H Rong M Zhang H Hou L Zhang C. Dysregulated GLUT1 may be involved in the pathogenesis of preeclampsia by impairing decidualization. Mol Cell Endocrinol (2022) 540:111509. doi: 10.1016/j.mce.2021.111509 34801669

[31] Ma Q Beal JR Bhurke A Kannan A Yu J Taylor RN. Extracellular vesicles secreted by human uterine stromal cells regulate decidualization, angiogenesis, and trophoblast differentiation. Proc Natl Acad Sci U S A (2022) 119(38):e2200252119. doi: 10.1073/pnas.2200252119 36095212 PMC9499590

[32] Frolova AI Moley KH. Quantitative analysis of glucose transporter mRNAs in endometrial stromal cells reveals critical role of GLUT1 in uterine receptivity. Endocrinology (2011) 152(5):2123–8. doi: 10.1210/en.2010-1266 PMC3075937 21343253

[33] von Wolff M Ursel S Hahn U Steldinger R Strowitzki T. Glucose transporter proteins (GLUT) in human endometrium: expression, regulation, and function throughout the menstrual cycle and in early pregnancy. J Clin Endocrinol Metab (2003) 88(8):3885–92. doi: 10.1210/jc.2002-021890 12915684

[34] Korgun ET Demir R Hammer A Dohr G Desoye G Skofitsch G. Glucose transporter expression in rat embryo and uterus during decidualization, implantation, and early postimplantation. Biol Reprod (2001) 65(5):1364–70. doi: 10.1095/biolreprod65.5.1364 11673251

[35] Jozaki K Tamura I Takagi H Shirafuta Y Mihara Y Shinagawa M. Glucose regulates the histone acetylation of gene promoters in decidualizing stromal cells. Reproduction (2019) 157(5):457–64. doi: 10.1530/rep-18-0393 30817321

[36] Jakson I Ujvari D Brusell Gidlöf S Lindén Hirschberg A. Insulin regulation of solute carrier family 2 member 1 (glucose transporter 1) expression and glucose uptake in decidualizing human endometrial stromal cells: an *in vitro* study. Reprod Biol Endocrinol (2020) 18(1):117. doi: 10.1186/s12958-020-00674-0 33218355 PMC7679983

[37] Chen W Lu S Yang C Li N Chen X He J. Hyperinsulinemia restrains endometrial angiogenesis during decidualization in early pregnancy. J Endocrinol (2019) 243(2):137–48. doi: 10.1530/joe-19-0127 31412315

[38] Chen Z Sandoval K Dean M. Endometrial glycogen metabolism during early pregnancy in mice. Mol Reprod Dev (2022) 89(9):431–40. doi: 10.1002/mrd.23634 PMC9796177 35842832

[39] Zhou WJ Yang HL Mei J Chang KK Lu H Lai ZZ. Fructose-1,6-bisphosphate prevents pregnancy loss by inducing decidual COX-2(+) macrophage differentiation. Sci Adv (2022) 8(8):eabj2488. doi: 10.1126/sciadv.abj2488 35196096 PMC8865779

[40] Vander Heiden MG Cantley LC Thompson CB. Understanding the warburg effect: the metabolic requirements of cell proliferation. Science (2009) 324(5930):1029–33. doi: 10.1126/science.1160809 PMC2849637 19460998

[41] Warburg O. On the origin of cancer cells. Science (1956) 123(3191):309–14. doi: 10.1126/science.123.3191.309 13298683

[42] Zuo RJ Gu XW Qi QR Wang TS Zhao XY Liu JL. Warburg-like glycolysis and lactate shuttle in mouse decidua during early pregnancy. J Biol Chem (2015) 290(35):21280–91. doi: 10.1074/jbc.M115.656629 PMC4571859 26178372

[43] Frolova AI O'Neill K Moley KH. Dehydroepiandrosterone inhibits glucose flux through the pentose phosphate pathway in human and mouse endometrial stromal cells, preventing decidualization and implantation. Mol Endocrinol (2011) 25(8):1444–55. doi: 10.1210/me.2011-0026 PMC3146244 21680659

[44] Tsai JH Schulte M O'Neill K Chi MM Frolova AI Moley KH. Glucosamine inhibits decidualization of human endometrial stromal cells and decreases litter sizes in mice. Biol Reprod (2013) 89(1):16. doi: 10.1095/biolreprod.113.108571 23718985 PMC4435226

[45] Critchley HOD Maybin JA Armstrong GM Williams ARW. Physiology of the endometrium and regulation of menstruation. Physiol Rev (2020) 100(3):1149–79. doi: 10.1152/physrev.00031.2019 32031903

[46] Mao C Liu X Guo SW. Decreased glycolysis at menstruation is associated with increased menstrual blood loss. Reprod Sci (2023) 30(3):928–51. doi: 10.1007/s43032-022-01066-y 36042151

[47] Pizer ES Kurman RJ Pasternack GR Kuhajda FP. Expression of fatty acid synthase is closely linked to proliferation and stromal decidualization in cycling endometrium. Int J Gynecol Pathol (1997) 16(1):45–51. doi: 10.1097/00004347-199701000-00008 8986532

[48] Sakoff JA Dunstan RH Murdoch RN. Uterine lipid alterations during early pseudopregnancy and following the artificial induction of decidualization by concanavalin a in QS mice. Mol Reprod Dev (1996) 44(1):93–102. doi: 10.1002/(sici)1098-2795(199605)44:1<93::Aid-mrd11>3.0.Co;2-1 8722697

[49] Rhee JS Saben JL Mayer AL Schulte MB Asghar Z Stephens C. Diet-induced obesity impairs endometrial stromal cell decidualization: a potential role for impaired autophagy. Hum Reprod (2016) 31(6):1315–26. doi: 10.1093/humrep/dew048 PMC4871191 27052498

[50] Downs SM Mosey JL Klinger J. Fatty acid oxidation and meiotic resumption in mouse oocytes. Mol Reprod Dev (2009) 76(9):844–53. doi: 10.1002/mrd.21047 PMC3995453 19455666

[51] Dunning KR Cashman K Russell DL Thompson JG Norman RJ Robker RL. Beta-oxidation is essential for mouse oocyte developmental competence and early embryo development. Biol Reprod (2010) 83(6):909–18. doi: 10.1095/biolreprod.110.084145 20686180

[52] Li N Gao R Chen X Liu X Ding Y He J. Carnitine palmitoyltransferase 1A is essential for decidualization in mice. Theriogenology (2022) 178:95–103. doi: 10.1016/j.theriogenology.2021.10.006 34837783

[53] Tsai JH Chi MM Schulte MB Moley KH. The fatty acid beta-oxidation pathway is important for decidualization of endometrial stromal cells in both humans and mice. Biol Reprod (2014) 90(2):34. doi: 10.1095/biolreprod.113.113217 24403548 PMC4435064

[54] Davidson KW Barry MJ Mangione CM Cabana M Caughey AB Davis EM. Aspirin use to prevent preeclampsia and related morbidity and mortality: US preventive services task force recommendation statement. JAMA (2021) 326(12):1186–91. doi: 10.1001/jama.2021.14781 34581729

[55] Gnecco JS Ding T Smith C Lu J Bruner-Tran KL Osteen KG. Hemodynamic forces enhance decidualization *via* endothelial-derived prostaglandin E2 and prostacyclin in a microfluidic model of the human endometrium. Hum Reprod (2019) 34(4):702–14. doi: 10.1093/humrep/dez003 PMC6443116 30789661

[56] Stadtmauer DJ Wagner GP. Single-cell analysis of prostaglandin E2-induced human decidual cell *in vitro* differentiation: a minimal ancestral deciduogenic signal†. Biol Reprod (2022) 106(1):155–72. doi: 10.1093/biolre/ioab183 PMC8757638 34591094

[57] Ye X. Lysophospholipid signaling in the function and pathology of the reproductive system. Hum Reprod Update (2008) 14(5):519–36. doi: 10.1093/humupd/dmn023 18562325

[58] Yamamoto Y Olson DM van Bennekom M Brindley DN Hemmings DG. Increased expression of enzymes for sphingosine 1-phosphate turnover and signaling in human decidua during late pregnancy. Biol Reprod (2010) 82(3):628–35. doi: 10.1095/biolreprod.109.081497 20007411

[59] Ye X Hama K Contos JJ Anliker B Inoue A Skinner MK. LPA3-mediated lysophosphatidic acid signalling in embryo implantation and spacing. Nature (2005) 435(7038):104–8. doi: 10.1038/nature03505 PMC1369590 15875025

[60] Jeng YJ Suarez VR Izban MG Wang HQ Soloff MS. Progesterone-induced sphingosine kinase-1 expression in the rat uterus during pregnancy and signaling consequences. Am J Physiol Endocrinol Metab (2007) 292(4):E1110–21. doi: 10.1152/ajpendo.00373.2006 17164439

[61] Spiegel S Milstien S. The outs and the ins of sphingosine-1-phosphate in immunity. Nat Rev Immunol (2011) 11(6):403–15. doi: 10.1038/nri2974 PMC3368251 21546914

[62] Aikawa S Kano K Inoue A Wang J Saigusa D Nagamatsu T. Autotaxin-lysophosphatidic acid-LPA(3) signaling at the embryo-epithelial boundary controls decidualization pathways. EMBO J (2017) 36(14):2146–60. doi: 10.15252/embj.201696290 PMC5509998 28588064

[63] Li Q Kannan A Das A Demayo FJ Hornsby PJ Young SL. WNT4 acts downstream of BMP2 and functions *via* β-catenin signaling pathway to regulate human endometrial stromal cell differentiation. Endocrinology. (2013) 154(1):446–57. doi: 10.1210/en.2012-1585 PMC3529366 23142810

[64] Large MJ Wetendorf M Lanz RB Hartig SM Creighton CJ Mancini MA. The epidermal growth factor receptor critically regulates endometrial function during early pregnancy. PLoS Genet (2014) 10(6):e1004451. doi: 10.1371/journal.pgen.1004451 24945252 PMC4063709

[65] Takano M Lu Z Goto T Fusi L Higham J Francis J. Transcriptional cross talk between the forkhead transcription factor forkhead box O1A and the progesterone receptor coordinates cell cycle regulation and differentiation in human endometrial stromal cells. Mol Endocrinol (2007) 21(10):2334–49. doi: 10.1210/me.2007-0058 17609436

[66] Huang X Withers BR Dickson RC. Sphingolipids and lifespan regulation. Biochim Biophys Acta (2014) 1841(5):657–64. doi: 10.1016/j.bbalip.2013.08.006 PMC3925463 23954556

[67] Ding NZ Qi QR Gu XW Zuo RJ Liu J Yang ZM. *De novo* synthesis of sphingolipids is essential for decidualization in mice. Theriogenology (2018) 106:227–36. doi: 10.1016/j.theriogenology.2017.09.036 29096270

[68] Chrostowski MK McGonnigal BG Stabila JP Padbury JF. Role of the l-amino acid transporter-1 (LAT-1) in mouse trophoblast cell invasion. Placenta (2010) 31(6):528–34. doi: 10.1016/j.placenta.2009.12.010 PMC2878872 20421131

[69] Zeng X Mao X Huang Z Wang F Wu G Qiao S. Arginine enhances embryo implantation in rats through PI3K/PKB/mTOR/NO signaling pathway during early pregnancy. Reproduction (2013) 145(1):1–7. doi: 10.1530/rep-12-0254 23081893

[70] Huang Z Wang TS Zhao YC Zuo RJ Deng WB Chi YJ. Cyclic adenosine monophosphate-induced argininosuccinate synthase 1 expression is essential during mouse decidualization. Mol Cell Endocrinol (2014) 388(1-2):20–31. doi: 10.1016/j.mce.2014.02.005 24556046

[71] Murthi P Wallace EM Walker DW. Altered placental tryptophan metabolic pathway in human fetal growth restriction. Placenta (2017) 52:62–70. doi: 10.1016/j.placenta.2017.02.013 28454699

[72] Cervenka I Agudelo LZ Ruas JL. Kynurenines: tryptophan's metabolites in exercise, inflammation, and mental health. Science (2017) 357(6349):eaaf9794. doi: 10.1126/science.aaf9794 28751584

[73] Wang PC Chen ST Hong ZK Li SY Yang ZS Quan S. Tryptophan and kynurenine stimulate human decidualization *via* activating aryl hydrocarbon receptor: short title: kynurenine action on human decidualization. Reprod Toxicol (2020) 96:282–92. doi: 10.1016/j.reprotox.2020.07.011 32781018

[74] Luo JM Zhang TT He YY Luo HN Hong YQ Yang ZM. Human chorionic gonadotropin-stimulated interleukin-4-Induced-1 (IL4I1) promotes human decidualization *via* aryl hydrocarbon receptor. Int J Mol Sci (2023) 24(4):3163. doi: 10.3390/ijms24043163 36834576 PMC9959871

[75] Tang L Xu XH Xu S Liu Z He Q Li W. Dysregulated gln-glu-α-ketoglutarate axis impairs maternal decidualization and increases the risk of recurrent spontaneous miscarriage. Cell Rep Med (2023) 101026:101026. doi: 10.1016/j.xcrm.2023.101026 PMC10213857 37137303

[76] Taghizadeh M Jamilian M Mazloomi M Sanami M Asemi Z. A randomized-controlled clinical trial investigating the effect of omega-3 fatty acids and vitamin e co-supplementation on markers of insulin metabolism and lipid profiles in gestational diabetes. J Clin Lipidol (2016) 10(2):386–93. doi: 10.1016/j.jacl.2015.12.017 27055970

[77] Huang J Xue M Zhang J Yu H Gu Y Du M. Protective role of GPR120 in the maintenance of pregnancy by promoting decidualization *via* regulation of glucose metabolism. EBioMedicine (2019) 39:540–51. doi: 10.1016/j.ebiom.2018.12.019 PMC6355327 30578080

[78] Chen Z, EY Xiong J Li W Chen X Li N. Dysregulated glycolysis underpins high-fat-associated endometrial decidualization impairment during early pregnancy in mice. Biochim Biophys Acta Mol Basis Dis (2023) 1869(4):166659. doi: 10.1016/j.bbadis.2023.166659 36740105

[79] Croxatto D Vacca P Canegallo F Conte R Venturini PL Moretta L. Stromal cells from human decidua exert a strong inhibitory effect on NK cell function and dendritic cell differentiation. PLoS One (2014) 9(2):e89006. doi: 10.1371/journal.pone.0089006 24586479 PMC3930605

[80] Lash GE Naruse K Robson A Innes BA Searle RF Robson SC. Interaction between uterine natural killer cells and extravillous trophoblast cells: effect on cytokine and angiogenic growth factor production. Hum Reprod (2011) 26(9):2289–95. doi: 10.1093/humrep/der198 21685139

[81] Krivonos MI Kh Khizroeva J Zainulina MS Eremeeva DR Selkov SA Chugunova A. The role of lymphocytic cells in infertility and reproductive failures in women with antiphospholipid antibodies. J Matern Fetal Neonatal Med (2022) 35(5):871–7. doi: 10.1080/14767058.2020.1732343 32098540

[82] Leonard S Murrant C Tayade C van den Heuvel M Watering R Croy BA. Mechanisms regulating immune cell contributions to spiral artery modification – facts and hypotheses – a review. Placenta (2006) 27 Suppl A:S40–6. doi: 10.1016/j.placenta.2005.11.007 16413937

[83] Dunn CL Critchley HO Kelly RW. IL-15 regulation in human endometrial stromal cells. J Clin Endocrinol Metab (2002) 87(4):1898–901. doi: 10.1210/jcem.87.4.8539 11932337

[84] Ye W Zheng LM Young JD Liu CC. The involvement of interleukin (IL)-15 in regulating the differentiation of granulated metrial gland cells in mouse pregnant uterus. J Exp Med (1996) 184(6):2405–10. doi: 10.1084/jem.184.6.2405 PMC2196382 8976195

[85] Ashkar AA Black GP Wei Q He H Liang L Head JR. Assessment of requirements for IL-15 and IFN regulatory factors in uterine NK cell differentiation and function during pregnancy. J Immunol (2003) 171(6):2937–44. doi: 10.4049/jimmunol.171.6.2937 12960317

[86] Brighton PJ Maruyama Y Fishwick K Vrljicak P Tewary S Fujihara R. Clearance of senescent decidual cells by uterine natural killer cells in cycling human endometrium. Elife (2017) 6:e31274. doi: 10.7554/eLife.31274 29227245 PMC5724991

[87] Zhang Y Wang Y Wang XH Zhou WJ Jin LP Li MQ. Crosstalk between human endometrial stromal cells and decidual NK cells promotes decidualization *in vitro* by upregulating IL−25. Mol Med Rep (2018) 17(2):2869–78. doi: 10.3892/mmr.2017.8267 PMC5783502 29257317

[88] Kammerer U Kruse A Barrientos G Arck PC Blois SM. Role of dendritic cells in the regulation of maternal immune responses to the fetus during mammalian gestation. Immunol Invest (2008) 37(5):499–533. doi: 10.1080/08820130802191334 18716936

[89] Blois SM Alba Soto CD Tometten M Klapp BF Margni RA Arck PC. Lineage, maturity, and phenotype of uterine murine dendritic cells throughout gestation indicate a protective role in maintaining pregnancy. Biol Reprod (2004) 70(4):1018–23. doi: 10.1095/biolreprod.103.022640 14681197

[90] Krey G Frank P Shaikly V Barrientos G Cordo-Russo R Ringel F. *In vivo* dendritic cell depletion reduces breeding efficiency, affecting implantation and early placental development in mice. J Mol Med (Berl) (2008) 86(9):999–1011. doi: 10.1007/s00109-008-0379-2 18575833

[91] Lessin DL Hunt JS King CR Wood GW. Antigen expression by cells near the maternal-fetal interface. Am J Reprod Immunol Microbiol (1988) 16(1):1–7. doi: 10.1111/j.1600-0897.1988.tb00169.x 3369615

[92] Mantovani A Biswas SK Galdiero MR Sica A Locati M. Macrophage plasticity and polarization in tissue repair and remodelling. J Pathol (2013) 229(2):176–85. doi: 10.1002/path.4133 23096265

[93] Houser BL Tilburgs T Hill J Nicotra ML Strominger JL. Two unique human decidual macrophage populations. J Immunol (2011) 186(4):2633–42. doi: 10.4049/jimmunol.1003153 PMC3712354 21257965

[94] Erlebacher A. Immunology of the maternal-fetal interface. Annu Rev Immunol (2013) 31:387–411. doi: 10.1146/annurev-immunol-032712-100003 23298207

[95] Tsai YC Tseng JT Wang CY Su MT Huang JY Kuo PL. Medroxyprogesterone acetate drives M2 macrophage differentiation toward a phenotype of decidual macrophage. Mol Cell Endocrinol (2017) 452:74–83. doi: 10.1016/j.mce.2017.05.015 28522271

[96] Care AS Diener KR Jasper MJ Brown HM Ingman WV Robertson SA. Macrophages regulate corpus luteum development during embryo implantation in mice. J Clin Invest (2013) 123(8):3472–87. doi: 10.1172/jci60561 PMC3726148 23867505

[97] Tilburgs T Claas FH Scherjon SA. Elsevier trophoblast research award lecture: unique properties of decidual T cells and their role in immune regulation during human pregnancy. Placenta (2010) 31 Suppl:S82–6. doi: 10.1016/j.placenta.2010.01.007 20106522

[98] Nancy P Tagliani E Tay CS Asp P Levy DE Erlebacher A. Chemokine gene silencing in decidual stromal cells limits T cell access to the maternal-fetal interface. Science (2012) 336(6086):1317–21. doi: 10.1126/science.1220030 PMC3727649 22679098

[99] Mor G Cardenas I Abrahams V Guller S. Inflammation and pregnancy: the role of the immune system at the implantation site. Ann N Y Acad Sci (2011) 1221(1):80–7. doi: 10.1111/j.1749-6632.2010.05938.x PMC3078586 21401634

[100] Tsuda H Michimata T Sakai M Nagata K Nakamura M Saito S. A novel surface molecule of Th2- and Tc2-type cells, CRTH2 expression on human peripheral and decidual CD4+ and CD8+ T cells during the early stage of pregnancy. Clin Exp Immunol (2001) 123(1):105–11. doi: 10.1046/j.1365-2249.2001.01422.x PMC1905966 11168006

[101] Wegmann TG Lin H Guilbert L Mosmann TR. Bidirectional cytokine interactions in the maternal-fetal relationship: is successful pregnancy a TH2 phenomenon? Immunol Today (1993) 14(7):353–6. doi: 10.1016/0167-5699(93)90235-d 8363725

[102] Liu ZK Wang RC Han BC Yang Y Peng JP. A novel role of IGFBP7 in mouse uterus: regulating uterine receptivity through Th1/Th2 lymphocyte balance and decidualization. PLoS One (2012) 7(9):e45224. doi: 10.1371/journal.pone.0045224 23028860 PMC3444470

[103] Saito S Nakashima A Shima T Ito M. Th1/Th2/Th17 and regulatory T-cell paradigm in pregnancy. Am J Reprod Immunol (2010) 63(6):601–10. doi: 10.1111/j.1600-0897.2010.00852.x 20455873

[104] Mjösberg J Berg G Jenmalm MC Ernerudh J. FOXP3+ regulatory T cells and T helper 1, T helper 2, and T helper 17 cells in human early pregnancy decidua. Biol Reprod (2010) 82(4):698–705. doi: 10.1095/biolreprod.109.081208 20018909

[105] Lim HW Lee J Hillsamer P Kim CH. Human Th17 cells share major trafficking receptors with both polarized effector T cells and FOXP3+ regulatory T cells. J Immunol (2008) 180(1):122–9. doi: 10.4049/jimmunol.180.1.122 18097011

[106] Du MR Guo PF Piao HL Wang SC Sun C Jin LP. Embryonic trophoblasts induce decidual regulatory T cell differentiation and maternal-fetal tolerance through thymic stromal lymphopoietin instructing dendritic cells. J Immunol (2014) 192(4):1502–11. doi: 10.4049/jimmunol.1203425 PMC3918863 24453244

[107] Li M Sun F Xu Y Chen L Chen C Cui L. Tim-3(+) decidual mφs induced Th2 and treg bias in decidual CD4(+)T cells and promoted pregnancy maintenance *via* CD132. Cell Death Dis (2022) 13(5):454. doi: 10.1038/s41419-022-04899-2 35550500 PMC9098864

[108] Wang S Sun F Li M Qian J Chen C Wang M. The appropriate frequency and function of decidual Tim-3(+)CTLA-4(+)CD8(+) T cells are important in maintaining normal pregnancy. Cell Death Dis (2019) 10(6):407. doi: 10.1038/s41419-019-1642-x 31138782 PMC6538701

[109] Wang S Chen C Li M Qian J Sun F Li Y. Blockade of CTLA-4 and Tim-3 pathways induces fetal loss with altered cytokine profiles by decidual CD4(+)T cells. Cell Death Dis (2019) 10(1):15. doi: 10.1038/s41419-018-1251-0 30622243 PMC6325160

[110] Wang S Zhu X Xu Y Zhang D Li Y Tao Y. Programmed cell death-1 (PD-1) and T-cell immunoglobulin mucin-3 (Tim-3) regulate CD4+ T cells to induce type 2 helper T cell (Th2) bias at the maternal-fetal interface. Hum Reprod (2016) 31(4):700–11. doi: 10.1093/humrep/dew019 26908841

[111] Wang SC Li YH Piao HL Hong XW Zhang D Xu YY. PD-1 and Tim-3 pathways are associated with regulatory CD8+ T-cell function in decidua and maintenance of normal pregnancy. Cell Death Dis (2015) 6(5):e1738. doi: 10.1038/cddis.2015.112 25950468 PMC4669692

[112] Li M Sun F Qian J Chen L Li D Wang S. Tim-3/CTLA-4 pathways regulate decidual immune cells-extravillous trophoblasts interaction by IL-4 and IL-10. FASEB J (2021) 35(8):e21754. doi: 10.1096/fj.202100142R 34191338

[113] He YY He XJ Guo PF Du MR Shao J Li MQ. The decidual stromal cells-secreted CCL2 induces and maintains decidual leukocytes into Th2 bias in human early pregnancy. Clin Immunol (2012) 145(2):161–73. doi: 10.1016/j.clim.2012.07.017 23069648

[114] Du MR Wang SC Li DJ. The integrative roles of chemokines at the maternal-fetal interface in early pregnancy. Cell Mol Immunol (2014) 11(5):438–48. doi: 10.1038/cmi.2014.68 PMC4197212 25109684

[115] Vacca P Vitale C Montaldo E Conte R Cantoni C Fulcheri E. CD34+ hematopoietic precursors are present in human decidua and differentiate into natural killer cells upon interaction with stromal cells. Proc Natl Acad Sci U S A (2011) 108(6):2402–7. doi: 10.1073/pnas.1016257108 PMC3038730 21248224

[116] Lu H Yang HL Zhou WJ Lai ZZ Qiu XM Fu Q. Rapamycin prevents spontaneous abortion by triggering decidual stromal cell autophagy-mediated NK cell residence. Autophagy. (2021) 17(9):2511–27. doi: 10.1080/15548627.2020.1833515 PMC8496707 33030400

[117] Shi JW Lai ZZ Yang HL Zhou WJ Zhao XY Xie F. An IGF1-expressing endometrial stromal cell population is associated with human decidualization. BMC Biol (2022) 20(1):276. doi: 10.1186/s12915-022-01483-0 36482461 PMC9733393

[118] Kusama K Yoshie M Tamura K Kodaka Y Hirata A Sakurai T. Regulation of decidualization in human endometrial stromal cells through exchange protein directly activated by cyclic AMP (Epac). Placenta. (2013) 34(3):212–21. doi: 10.1016/j.placenta.2012.12.017 23352189

[119] Jin X Cui L Zhao W Li X Liu L Li Y. Decidualization-derived cAMP regulates phenotypic and functional conversion of decidual NK cells from CD56(dim)CD16(-) NK cells. Cell Mol Immunol (2021) 18(6):1596–8. doi: 10.1038/s41423-021-00675-y PMC8166858 33785840

[120] Zeng S Liang Y Lai S Bi S Huang L Li Y. TNFα/TNFR1 signal induces excessive senescence of decidua stromal cells in recurrent pregnancy loss. J Reprod Immunol (2023) 155:103776. doi: 10.1016/j.jri.2022.103776 36495656

[121] Hu WT Huang LL Li MQ Jin LP Li DJ Zhu XY. Decidual stromal cell-derived IL-33 contributes to Th2 bias and inhibits decidual NK cell cytotoxicity through NF-κB signaling in human early pregnancy. J Reprod Immunol (2015) 109:52–65. doi: 10.1016/j.jri.2015.01.004 25712540

[122] Valero-Pacheco N Tang EK Massri N Loia R Chemerinski A Wu T. Maternal IL-33 critically regulates tissue remodeling and type 2 immune responses in the uterus during early pregnancy in mice. Proc Natl Acad Sci U S A (2022) 119(35):e2123267119. doi: 10.1073/pnas.2123267119 35994660 PMC9436313

[123] Jiang L Cao D Yeung WSB Lee KF. Single-cell RNA-sequencing reveals interactions between endometrial stromal cells, epithelial cells, and lymphocytes during mouse embryo implantation. Int J Mol Sci (2022) 24(1):213. doi: 10.3390/ijms24010213 36613656 PMC9820401

[124] Huang HL Yang HL Lai ZZ Yang SL Li MQ Li DJ. Decidual IDO(+) macrophage promotes the proliferation and restricts the apoptosis of trophoblasts. J Reprod Immunol (2021) 148:103364. doi: 10.1016/j.jri.2021.103364 34482001

[125] Grasso E Paparini D Agüero M Mor G Pérez Leirós C Ramhorst R. VIP Contribution to the decidualization program: regulatory T cell recruitment. J Endocrinol (2014) 221(1):121–31. doi: 10.1530/joe-13-0565 24492467

[126] Grasso E Gori S Paparini D Soczewski E Fernández L Gallino L. VIP Induces the decidualization program and conditions the immunoregulation of the implantation process. Mol Cell Endocrinol (2018) 460:63–72. doi: 10.1016/j.mce.2017.07.006 28689770

[127] Menkhorst EM Van Sinderen ML Rainczuk K Cuman C Winship A Dimitriadis E. Invasive trophoblast promote stromal fibroblast decidualization *via* profilin 1 and ALOX5. Sci Rep (2017) 7(1):8690. doi: 10.1038/s41598-017-05947-0 28821715 PMC5562808

[128] Zhang XY Shen HH Qin XY Wang CJ Hu WT Liu SP. IL-27 promotes decidualization *via* the STAT3-ESR/PGR regulatory axis. J Reprod Immunol (2022) 151:103623. doi: 10.1016/j.jri.2022.103623 35430461

[129] Zhou X Cao Y Zhou M Han M Liu M Hu Y. Decreased CD44v3 expression impairs endometrial stromal cell proliferation and decidualization in women with recurrent implantation failure. Reprod Biol Endocrinol (2022) 20(1):170. doi: 10.1186/s12958-022-01042-w 36527033 PMC9756673

[130] Zhu RH Dai FF Yang DY Liu SY Zheng YJ Wu ML. The mechanism of insulin-like growth factor II mRNA-binging protein 3 induce decidualization and maternal-fetal interface cross talk by TGF-β1 in recurrent spontaneous abortion. Front Cell Dev Biol (2022) 10:862180. doi: 10.3389/fcell.2022.862180 35465321 PMC9023862

[131] Ma Q Beal JR Song X Bhurke A Bagchi IC Bagchi MK. Extracellular vesicles secreted by mouse decidual cells carry critical information for the establishment of pregnancy. Endocrinology. (2022) 163(12):bqac165. doi: 10.1210/endocr/bqac165 36219207 PMC9761388

[132] Teklenburg G Salker M Heijnen C Macklon NS Brosens JJ. The molecular basis of recurrent pregnancy loss: impaired natural embryo selection. Mol Hum Reprod (2010) 16(12):886–95. doi: 10.1093/molehr/gaq079 20847090

[133] Rabaglino MB Post Uiterweer ED Jeyabalan A Hogge WA Conrad KP. Bioinformatics approach reveals evidence for impaired endometrial maturation before and during early pregnancy in women who developed preeclampsia. Hypertension (2015) 65(2):421–9. doi: 10.1161/hypertensionaha.114.04481 PMC4290371 25421975

[134] Founds SA Conley YP Lyons-Weiler JF Jeyabalan A Hogge WA Conrad KP. Altered global gene expression in first trimester placentas of women destined to develop preeclampsia. Placenta. (2009) 30(1):15–24. doi: 10.1016/j.placenta.2008.09.015 19027158 PMC2667803

